# The Pacific Oyster Mortality Syndrome, a Polymicrobial and Multifactorial Disease: State of Knowledge and Future Directions

**DOI:** 10.3389/fimmu.2021.630343

**Published:** 2021-02-18

**Authors:** Bruno Petton, Delphine Destoumieux-Garzón, Fabrice Pernet, Eve Toulza, Julien de Lorgeril, Lionel Degremont, Guillaume Mitta

**Affiliations:** ^1^ Ifremer, LEMAR UMR 6539, UBO/CNRS/IRD/Ifremer, Argenton-en-Landunvez, France; ^2^ IHPE, Université de Montpellier, CNRS, Ifremer, Université de Perpignan Via Domitia, Montpellier, France; ^3^ Ifremer, SG2M, LPGMM, La Tremblade, France

**Keywords:** Pacific oyster mortality syndrome, polymicrobial disease, multifactorial disease, *Crassostrea gigas*, OsHV-1, opportunistic bacterial pathogens

## Abstract

The Pacific oyster (*Crassostreae gigas*) has been introduced from Asia to numerous countries around the world during the 20th century. *C. gigas* is the main oyster species farmed worldwide and represents more than 98% of oyster production. The severity of disease outbreaks that affect *C. gigas*, which primarily impact juvenile oysters, has increased dramatically since 2008. The most prevalent disease, Pacific oyster mortality syndrome (POMS), has become panzootic and represents a threat to the oyster industry. Recently, major steps towards understanding POMS have been achieved through integrative molecular approaches. These studies demonstrated that infection by Ostreid herpesvirus type 1 µVar (OsHV-1 µvar) is the first critical step in the infectious process and leads to an immunocompromised state by altering hemocyte physiology. This is followed by dysbiosis of the microbiota, which leads to a secondary colonization by opportunistic bacterial pathogens, which in turn results in oyster death. Host and environmental factors (*e.g.* oyster genetics and age, temperature, food availability, and microbiota) have been shown to influence POMS permissiveness. However, we still do not understand the mechanisms by which these different factors control disease expression. The present review discusses current knowledge of this polymicrobial and multifactorial disease process and explores the research avenues that must be investigated to fully elucidate the complexity of POMS. These discoveries will help in decision-making and will facilitate the development of tools and applied innovations for the sustainable and integrated management of oyster aquaculture.

## Introduction

Aquaculture is one of the fastest-growing food industries, representing more than 50% of worldwide seafood production (1). Mollusk aquaculture, in which oysters are the most important taxonomic group (by volume), has become one of the largest animal food-producing industries.

The Pacific oyster *Crassostrea gigas* was introduced from Asia to numerous countries throughout the world (*e.g.* Canada, the USA, Brazil, Australia, New Zealand, Chile, Mexico, Argentina, South Africa, Namibia, and numerous European countries including France) during the 20th century (2). Worldwide production of *C. gigas* is estimated at 4.7 million tons per year (3). China is, by far, the leading producer, followed by South Korea, Japan, and France.

For decades, *C. gigas* has suffered from mortality due to disease (4), but the severity of these outbreaks has increased dramatically since 2008. These outbreaks affect the juvenile stages of the oyster, killing more than 35% of the cultivated and natural oysters in France every year (5, 6). The corresponding syndrome, referred to as Pacific oyster mortality syndrome (POMS) (7), has become panzootic; it is observed in all the coastal regions of Franc, and in numerous other countries worldwide (8). The ecological and economic consequences of POMS can be dramatic (9), and POMS represents a threat to the worldwide oyster industry.

POMS is a disease with complex etiology. Research efforts have revealed a series of factors that contribute to the disease, including interactions between infectious agents and seawater temperature, oyster genetics, food availability, and oyster growth (9–17).

In this review, we present the research performed in the past decade that has contributed to a better understanding of POMS. These studies have deciphered the polymicrobial nature of the disease. Although this is a first and important step, POMS remains an incompletely understood multifactorial disease that is controlled by a series of host and environmental factors. It is crucial that POMS be fully deciphered by the determination of which factors control disease expression, how these factors control disease expression, the weight of these different factors, and their putative synergistic and antagonistic effects. Only once this work is complete will we completely understand POMS and be able to propose solutions to ensure the durability of the oyster industry.

## POMS is a Polymicrobial Disease

Dramatic increases in oyster mortality (observed since 2008) have been found to coincide with the recurrent detection of *Ostreid herpesvirus* (OsHV-1) variants in moribund oysters in France (17–19) and worldwide (9, 20–23). Because of this, research efforts have historically focused on the viral etiology of POMS and a series of diagnostic assays such as PCR, real-time PCR, and *in situ* hybridization have been developed to detect OsHV-1 (18, 19, 24–26). Nevertheless, the involvement of other etiological agents was suspected (15). In particular, bacterial strains of the genus *Vibrio* had been shown to be associated with POMS (27, 28). However, the role of these different putative pathogens has been tested in isolation, using experimental systems (bacterial or viral filtrate injections) that did not reproduce the natural route of infection. Consequently, the complex POMS infectious process remained misunderstood.

Several breakthroughs have advanced our understanding of the complexity of POMS. The development of an ecologically realistic model of infection was the first major progress (16, 29). Briefly, this method of infection uses pathogen-free oysters that are reared in bio-secured conditions from birth to 3 months of age. Some of these pathogen-free oysters are then naturally infected in the field to become “donors,” while the remaining oysters are maintained in bio-secured conditions as “recipients.” Recipients are subsequently exposed to donor oysters through cohabitation. This method retains the complexity of the infectious environment (OsHV-1 and populations of virulent bacteria) and mimics the natural route of infection (16, 29). This method also allows simultaneous triggering of disease in all recipients and parallels the dynamics of the disease through time on oysters that are phased according to the infectious process in which they are engaged. A second important breakthrough was the use of oyster biparental families selected for higher disease resistance (10). By reducing genetic diversity, which is particularly high in oysters, the disease dynamics can be monitored in oysters that have contrasting phenotypes (susceptible or resistant) during pathogen challenge. A final breakthrough was the use of integrative molecular approaches that allowed the observation of the dynamics of the host response and the microbiota, including putative pathogens, in the same experimental framework.

**Figure 1:**
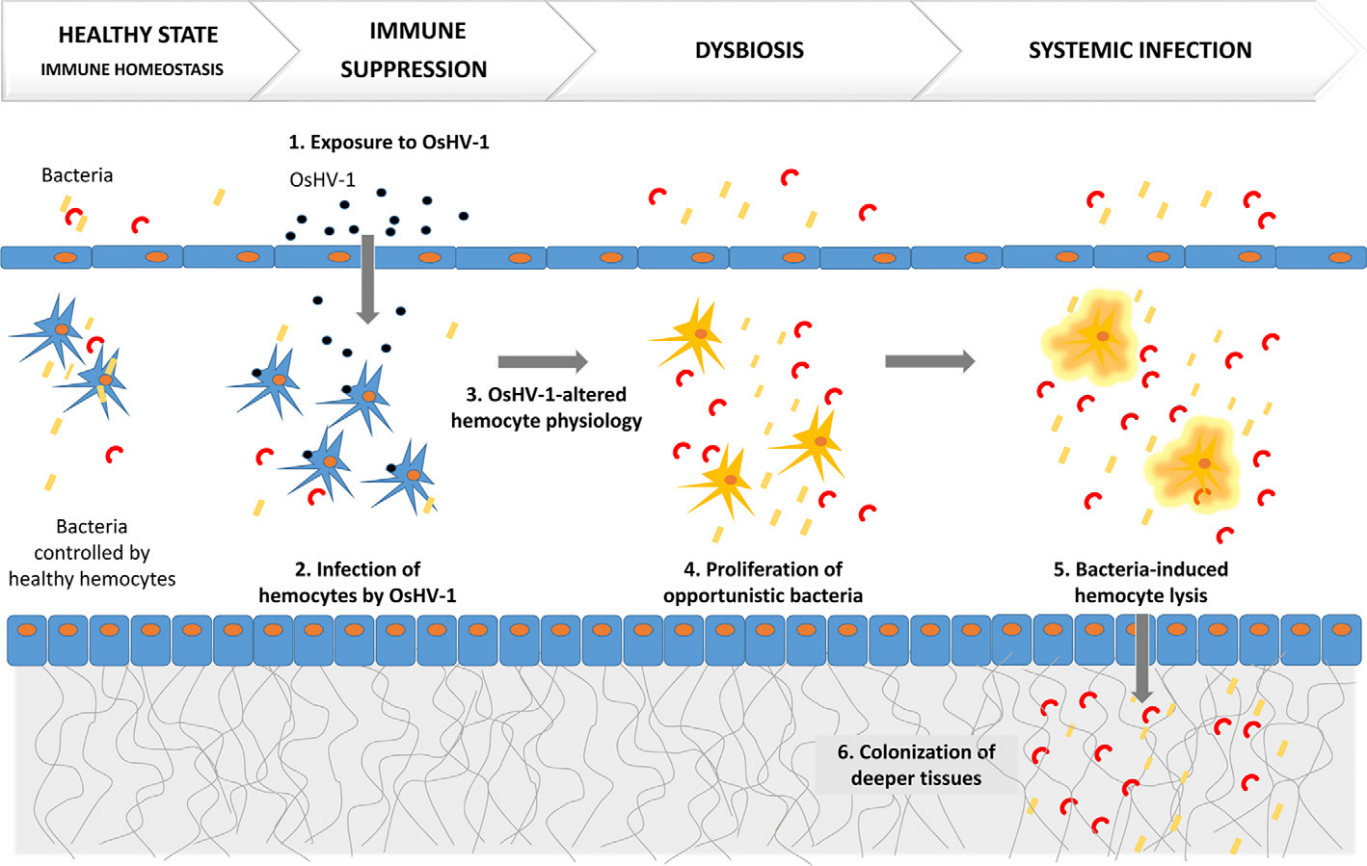
POMS is a polymicrobial disease induced by a primary infection by OsHV-1, which alters hemocyte physiology. This is followed by secondary bacteremia that leads to oyster death.

One recent study that combined an ecologically realistic model of infection, the use of susceptible and resistant oyster families, and integrative molecular approaches (dual RNAseq, 16S rDNA metabarcoding, and histology) deciphered the mechanism of POMS (30). This study demonstrated that POMS is a polymicrobial disease (**Figure 1**). Early gill damages, infection of hemocytes by OsHV-1, and bacterial colonization both inside and outside the gill tissues were evidenced in susceptible oysters only (30). This study revealed that infection by OsHV-1 is the first critical step in POMS and leads to an immunocompromised state by infecting and altering hemocyte physiology (30). This immunosuppression subsequently evolves towards bacteremia, which involves a series of opportunistic bacteria (30). This bacteremia leads to oyster death. Indeed, POMS requires both OsHV-1 and opportunistic bacteria: preventing either viral replication or bacterial proliferation with poly-IC injection or antibiotics, respectively, blocks the infectious process and prevents mortality (30).

### Primary Viral Infection

Infection by OsHV-1 µVar is the first event in the infectious process of POMS, and intense viral replication is a prerequisite for the development of the disease. The inability of susceptible oysters to control viral replication during POMS is associated with a strong, but late, antiviral response (30). Concomitant with the intense replication of the virus, oysters intensively express several genes that encode endogenous Inhibitors of Apoptosis (IAPs) (30). Although the pathway by which OsHV-1 could control endogenous IAP expression is unknown, such a mechanism has been described in a human Gammaherpesvirus, Epstein–Barr virus, which is able to increase the expression of IAP-2 to inhibit apoptosis (31). Remarkably, intense OsHV-1 replication is also associated with high expression of exogenous IAPs of viral origin (30). Such viral proteins, which are of the BIR family, are known to have anti-apoptotic activities that favor viral replication (32). These results suggest that both endogenous and exogenous anti-apoptotic processes, which are strongly activated in susceptible oysters, play a key role in the success of OsHV-1 infection (30).

Oyster immune cells, called hemocytes, are targeted by OsHV-1 (30, 33). Infection of hemocytes by OsHV-1 impacts hemocyte physiology and impairs the expression of antimicrobial peptides (30), either directly (through transcriptional regulation) or indirectly (through the induction of cell death or lysis processes) (33).

Since the description of the first OsHV-1 µVar genotype in 2010 (17), increasing NGS sequencing data have revealed the diversity of OsHV-1 µVar genotypes (17, 22, 23, 34, 35). This observation, similar to observations made in a number of RNA and DNA viruses (36–39), raises the question of what impact this genetic diversity has on the fitness of the virus and the consequences of the disease. Many viruses produce diverse genetically linked variants that can be defined as viral populations. These populations are maintained by mutation-selection equilibrium (40, 41), and have the potential to generate beneficial interactions and cooperation which increase viral fitness and adaptability to the host (42–45).

A recent study investigated these possibilities in OsHV-1 (46). Different biparental families of oysters were confronted with two different infectious environments. Because susceptibility to POMS can differ not only between families within the same environment, but also within the same family between the two environments, viral diversity was analyzed between families and environments (46). This analysis revealed distinct viral populations in the two infectious environments (46). Moreover, the different oyster families were infected by distinct viral populations within the same infectious environment (46). These results suggest that there are co-evolutionary processes at play between OsHV-1 μVar, and that oyster populations have selected for a diversity of viral populations that could, in turn, facilitate viral adaptation to various environments and various host genotypes.

### Secondary Bacterial Infection

Until recently, the bacterial component of POMS has mainly been studied using culture-based approaches. These approaches revealed a clear association between some *Vibrio* species and POMS, which has led to extensive characterization of the roles and contributions of *Vibrio* bacteria to oyster mortality.

Using pathogen-free oyster spats and field-based approaches, Le Roux and collaborators characterized the population structure of *Vibrio* species found in naturally infected oysters during POMS episodes on the French Atlantic coast. Members of the *Splendidus* clade (*e.g. V. tasmaniensis, V. splendidus, V. cyclitrophicus, V. harveyi, V. aestuarianus*, and *V. crassostreae*) have been systematically isolated from diseased juvenile oysters, as have (to a lesser extent) *V. harveyi* and *V. aestuarianus*, which fall outside the clade (17, 27, 47). Bruto et al. demonstrated that the *Vibrio* population structure is seasonal and varies in oysters affected by POMS (27). Notably, *Vibrio* of the *Splendidus* clade are present in healthy oysters when no mortalities occur (27), but they only express low to moderate pathogenic potential in these circumstances (48). *V. crassostreae* is predominant during mortalities and is almost exclusively associated with oyster tissues (27). *V. crassostreae* has been shown to replace the resident *Vibrio* community during a POMS episode (49).

Some factors that contribute to *Vibrio* virulence in oysters have been discovered in species that exhibit pathogenic potential in experimental infections (*e.g.* bacteria injected in the adductor muscle). These factors have mostly been described in *V. crassostreae* and *V. tasmaniensis* [a facultative intracellular pathogen of oyster hemocytes (50)] isolated from the Atlantic during POMS episodes (27, 28, 49–52). Bruto et al. (28) found that the r5.7 gene is required for virulence and is ancestral in the Splendidus clade. The R5.7 protein itself is not cytotoxic (28); however, to mediate cytotoxicity, R5.7-expressing *V. crassostreae* requires physical contact with hemocytes (51). Interestingly, upon the loss of the ancestral r5.7 gene, *V. tasmaniensis* has acquired a type 6 secretion system (T6SS) on chromosome 1. This T6SS intracellularly delivers cytotoxic effectors to oyster hemocytes (51). These findings show that *Vibrio* cytotoxicity is a key determinant of oyster colonization that allows *Vibrio* to escape from potent cellular defenses and cause systemic infection (51). Furthermore, these findings show that distinct molecular determinants can confer similar dampening of host immune defenses in *Vibrio* species associated with POMS.

To date, *Vibrio* is the only bacterial group that has been studied in detail as an etiological agent of POMS, in part because *Vibrio* species are readily cultured and amenable to functional studies. Other bacterial groups (*e.g. Arcobacter* and *Shewanella*) have been associated with oyster mortality (30, 53, 54). However, their particular contributions to, and potential cooperation with other bacterial communities that participate in, fatal dysbiosis remain unknown, largely due to technical limitations.

Recently, we studied the structure of bacterial communities (as determined by 16S metabarcoding) and the functions expressed by different bacterial genera (as determined by metatranscriptomics) in different oyster biparental families submitted to different infectious environment (55). Five bacterial genera (*Arcobacter, Marinobacterium, Marinomonas, Vibrio*, and *Pseudoalteromonas)* that colonize oysters during POMS were remarkably consistent between families and environments. These genera are referred to as the POMS pathobiota.

The core functions identified by metatranscriptomics in POMS pathobiota reveal the importance of general metabolism and adaptation to stress (imposed by host defenses) in the success of colonization (55). Beyond general metabolism, detoxifying enzymes (such as alkylhydroxyperoxydases) involved in the resistance to or tolerance of host immune defenses are expressed by all successful colonizers. Most of the successful genera also express enzymes required for tolerance/resistance to oxidative stress. Reactive Oxygen Species (ROS) and Reactive Nitrogen Species (RNS) are major players at the oyster-microbiota interface (56). Thus, it is not surprising that successful colonizers express ROS/RNS detoxifying enzymes during oyster colonization.

A number of genus-specific functions that likely confer cross-benefits at the community level are also expressed during pathogenesis (55). These include genes related to metal homeostasis (siderophore production), iron uptake, and virulence (*e.g.* T6SS), all of which contribute to suppression of oyster cellular defenses (51, 57). The expression of these genes is likely beneficial to the entire bacterial community. From a metabolic point of view, cross-benefits are best exemplified by sulfur metabolism, a pathway which is non-redundantly encoded by five different genera. This suggests that sulfur cycling is a core property of the colonizing microbiota as a whole, rather than of a single genus. Such metabolic interdependence between, and cooperation of, microbial communities is likely a key determinant of the structure of the POMS pathobiota, and may dictate its conservation across distinct environments and oyster genetic backgrounds.

Many questions remain to be studied, including how *Vibrio* and/or other bacterial genera collaborate with OsHV-1 to kill oysters. It is known that the molecular manipulation of hemocyte molecular responses by OsHV-1 (i.e. the inhibition of antimicrobial peptide expression) is a key determinant of bacterial colonization, without which *Vibrio* species fail to efficiently colonize oysters and express their pathogenic potential (30). It is also known that the dampening of oyster cellular defenses by *Vibrio* (through active hemocyte lysis), or possibly by other pathogenic bacteria, is key in the development of systemic infection (51). Because hemocytes are composed of diverse populations of cells that play different roles in infection control, better identification of the cell populations targeted by each POMS-associated pathogen is required to understand the polymicrobial nature of POMS at the cellular and molecular scales.

## POMS is a Multifactorial Disease

The risk of POMS outbreaks has been linked to subtle interactions between not only host and pathogens, but also environmental factors. Thus, POMS is a not only a polymicrobial disease, but also a multifactorial disease. In this review, we focus on the five main environmental and host factors associated with disease expression: i) temperature, ii) oyster genetics, iii) oyster age, iv) oyster energetic status, and v) microbiota.

### Temperature

**Figure 2:**
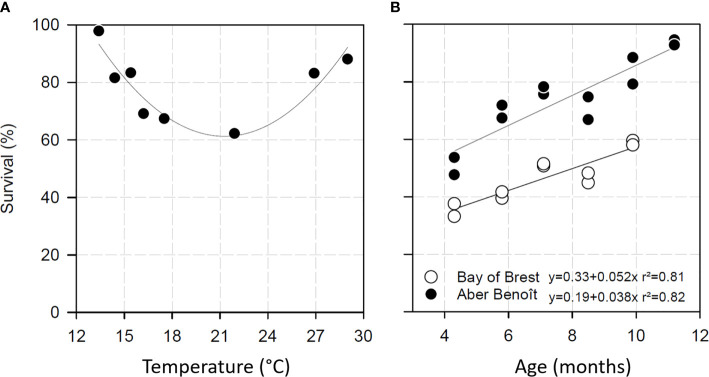
Survival of Pacific oysters exposed to POMS according to temperature **(A)** and age **(B)**. **(A)** *C. gigas* survival after exposition to POMS in mesocosm. Oyster mortalities after 16 days of exposition were maximal at temperatures ranging from 16.2 and 21.9°C. Lower and upper temperatures diminished POMS permissiveness. Data were extracted from (17). **(B)** Survival of Pacific oysters exposed to POMS according to their age in two Atlantic coast locations (Aber Benoît and Bay of Brest). The data are modified from (65).

Temperature increases linked to climate warming are a major driver of disease outbreaks (58). In different invertebrate–pathogen interactions, temperature has been found to affect the outcome of the interaction by influencing host and/or pathogen physiology (59–63). A permissive temperature range for POMS has been clearly identified, specifically, 16°C to 24°C (5, 6, 64). The same trend was observed in mesocosm experiments in which recipient oysters were confronted to disease donors at different temperature [**Figure 2A**, (16)].

Although higher temperatures decrease oyster susceptibility to POMS, viral infectivity is quite similar to infectivity at permissive temperatures (66). This reinforces the hypothesis that other factors (*e.g.* bacterial virulence or oyster physiology) could be influenced by high temperatures and thereby affect POMS permissiveness. Although the effect of high temperatures on bacterial virulence remains to be investigated, a recent work demonstrated that oyster immunity is modulated, and apoptotic processes induced, by high temperatures, which could explain the observed decrease of permissiveness (11).

With respect to lower temperatures, diseased oysters exposed to low temperature (13°C) over the course of 40 days exhibited no mortality, were negative for OsHV-1, and did not transmit the disease to healthy oysters (16). The mechanisms by which low temperatures affect POMS permissiveness remain to be investigated.

### Oysters Genetics

Some oysters, particularly those able to limit infection by OsHV-1, are resistant to POMS. Such resistant oysters were first described in field challenges (67), and subsequently in laboratory challenges, as exhibiting a 3 log of decrease in viral DNA copies (30, 68, 69). Genetic studies of oyster resistance have revealed a significant additive genetic component of survival during OsHV-1 infection (10, 70–72). Over the past decade, many oyster genomic tools [including a reference genome (73) and SNP arrays (74)] have been developed to expand the study of the genetic underpinnings of *C. gigas* resistance to OsHV-1 infection.

The results of genome-wide association studies in juvenile oysters experimentally challenged with OsHV-1 and genotyped using a high-density linkage map constructed for the Pacific oyster (75) suggest that OsHV-1 resistance is polygenic in nature. These studies also highlighted that region of linkage group 6 contains a significant QTL which affects host resistance (75). Several SNPs, associated with survival and/or viral load, were located in several genes that encode RAN Binding Protein 9, a Coronin and Myo10 (an actin motor protein). However, the specific roles of these genes in the resistance process remain to be elucidated.

A recent transcriptomic study on several oyster biparental families that exhibited different susceptibilities to POMS revealed that the early induction of genes involved in antiviral defense is a hallmark of resistant oyster families (30). However, the specific genetic components responsible for this early induction remain unidentified. To identify putative transcriptomic determinants associated with POMS resistance, basal transcriptomes (no disease challenge) of three resistant oyster families were compared to the basal transcriptomes of three susceptible families (76). POMS resistant oysters showed constitutive differences in the expression of genes involved in stress responses, protein modification, maintenance of DNA integrity and DNA repair, and immune and antiviral pathways. Similarities and differences between molecular pathways were observed among the resistant families. For example, several genes related to the TLR-NF-*κ*B, JAK-STAT, and STING-RLR pathways were identified (76). Among them, only one transcript was overrepresented in the three resistant families (76). This transcript corresponded to an endosomal Toll-like Receptor that displays similarities to TLR 13, which can act as a sensor of viral and bacterial RNA in the TLR-NF-*κ*B signaling pathway (77, 78). This gene is particularly interesting because its function could explain how these resistant oyster families detect viral infection early, which allows them to mount a more rapid and efficient antiviral response (30). Given this putative antiviral role, TLR 13 represents a good candidate for future study.

Overall, the results of these studies suggest that the POMS resistance process is polygenic and varies somewhat with oyster genotype.

### Oyster Age

A series of studies have reported that POMS-induced mortality rates are lower in adult oysters than in spat and juvenile oysters (13, 20, 79). The experimental design of these studies, however, did not allow researchers to disentangle the effect of selection on resistance and age. In fact, the adult oysters analyzed in these studies had survived one or more POMS events, and thus might have been selected for resistance to POMS over time.

To further investigate the effect of oyster age on mortality, healthy oysters at different developmental stages (juveniles and adults) were exposed to the Marennes-Oléron Bay (France) infectious environment (80). In this study, oyster age was found to be inversely correlated with mortality. This result was confirmed both in the same infectious environment (10) and in another infectious environment (Brest Bay, France) (65). In the Brest Bay study, healthy oysters of different ages were simultaneously confronted with the same infectious environment. Most oysters appear to become resistant to POMS with age (**Figure 2B**), and resistance appears to be acquired after 24 months (10, 65). It is expected that the maturation of the immune system underlies the acquisition of resistance; however, the underlying molecular mechanisms remain to be investigated.

### Food Availability, Growth, and Energy Reserves

Food availability is known to influence disease risk and outcome in two ways. First, food availability improves the physiological condition of the host and lowers their susceptibility to infectious disease, which reflects a tradeoff between immunity and other functions [*e.g.* (81, 82)]. Second, food scarcity limits the resources available to the pathogen, because it slows the growth and metabolism of the host, which the pathogen depends on in order to proliferate (83–87). Therefore, food availability can have both positive and negative effects on the severity of infectious diseases.

**Figure 3:**
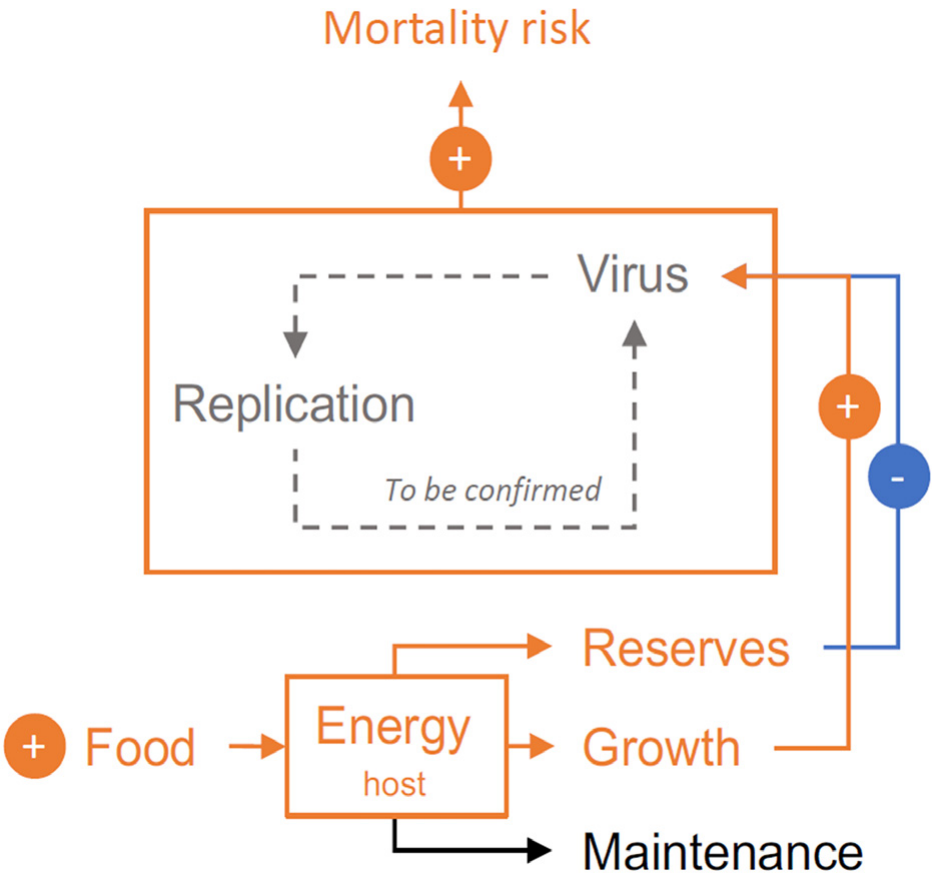
Food availability both enables mortality by increasing oyster growth (and possibly viral replication), and limits mortality by increasing oyster energy. The net effect of increased food availability is an increased risk of mortality in oysters exposed to POMS. Orange, blue and, black lines indicate positive, negative, and neutral feedback, respectively. Gray dashed line indicates hypothetical mechanism.

Previous studies have demonstrated that fed oysters are at a higher risk of death than starved oysters (88, 89). Paradoxically, the energy reserves of oysters, which reflect food availability, are associated with increased resistance or tolerance to OsHV-1 (13, 64, 90). In light of these results, Pernet et al. (14) investigated how food availability, growth rate, and energy reserves drive the outcome of POMS. Briefly, the authors selected fast- and slow-growing oysters and exposed these oysters to high and low food rations. The oysters were evaluated for energy reserves, challenged with POMS, and monitored for survival. The study found that although higher food levels and oyster growth are associated with an increased mortality risk, higher energy reserves are associated with decreased mortality risk (14). Thus, food availability both enables mortality by increasing oyster growth and limits mortality by increasing energy reserves (**Figure 3**). This study clarifies the impact of food resources on disease susceptibility and suggests how host physiological condition could mitigate epidemics (14).

The specific mechanisms by which food availability, growth rate, and energy reserves modulate oyster susceptibility to infection remain to be elucidated. We hypothesize that the positive association between food level and mortality risk reflects increased host growth and metabolic rate, which in turn amplifies pathogen replication. Like other viruses, OsHV-1 uses host cellular machinery to replicate (91, 92), thus stimulation of host cellular growth could amplify viral gene expression and replication. However, there is currently no evidence that demonstrates increased viral replication in response to increased food availability and host growth rate. Furthermore, we cannot exclude the possibility that food availability, growth rate, and energy reserves act on susceptibility to secondary bacterial infection. Finally, the lower mortality risk observed at low food levels may also reflect induced autophagy, an evolutionarily conserved cell recycling process that is activated in response to stress (including starvation) (93). Autophagy also controls microbial infections, both through direct destruction of the pathogen, and indirectly as a key mediating factor in host innate and adaptive immunity (93). The autophagy pathway is functional in oysters and could explain why starvation reduces mortality during OsHV-1 infection (89). Further investigations are needed to assess whether and how food levels, growth, and energy reserves control viral proliferation, bacteremia, and/or autophagy.

### Microbiota

Several independent studies used 16S metabarcoding to investigate uncultivable bacterial microbiota. These studies identified shifts in the composition of the microbiota community (dysbiosis) associated with POMS. In a large-scale analysis of the microbiota of diseased oysters from three different sites in Europe, Lasa and colleagues identified dysbiosis in OsHV-1 positive oysters. This dysbiosis was characterized by the emergence of a pathobiota composed of opportunistic pathogens (including *Vibrio* and *Arcobacter* species) (54). In the most integrative study of POMS to date, ecologically realistic infection demonstrated that bacterial dysbiosis is subsequent to viral infection, that viral infection leads to antibacterial defense impairment, and that this impairment allows opportunistic pathogens to colonize oysters (30).

In addition, oyster genotype-specific microbial associations have been identified between genetically differentiated oyster beds especially for the rare phylotype assembling (94). This association disappeared under environmental stress. Constitutive differences in microbiota have also been identified in 35 healthy oyster breeds displaying different levels of resistance to POMS (95). Operational Taxonomic Units (OTUs) of the *Photobacterium, Vibrio, Aliivibrio, Streptococcus*, and *Roseovarius* genera were significantly associated with the most susceptible oyster families, as was a higher background load of rare *Vibrio* OTUs in the healthy state. This suggests that these susceptible families exhibit a decreased immune response to these pathogens (95).

In another study, the Mycoplasmataceae, Rhodospirillaceae, and Vibrionaceae bacterial families, as well as the *Photobacterium* genus, were associated with susceptible oyster families (96). The proportion of specific taxa, including Cyanobacteriaceae, Colwelliaceae, and Rhodobacteraceae, was significantly higher in resistant oyster families that survived POMS after transplant into the field during an infectious period. Resistant oysters also displayed a higher evenness of the bacterial colonizers, suggesting that a decrease in microbial diversity may be associated with a loss of microbiota function, which could allow colonization by opportunistic pathogens (96).

This research raises questions about the role of microbiota composition and stability in oyster health and susceptibility to POMS. Whether differences in bacterial community composition and dynamics are responsible for different disease resistance levels, or are only related to the host-genotype specificity of the microbiota, remains to be elucidated. Future studies are needed to identify the relative contributions of the oyster microbiota to disease outcome, including studies of direct interactions with pathobiota and possible immune stimulation of the host.

## Conclusions and Perspectives

Determination of the mechanism of POMS pathogenesis and identification of host, pathogen, and environmental factors that influence POMS are important achievements. However, these are only initial steps in the development of new approaches to counter POMS and its devastating effects on the oyster industry.

The research discussed above, along with future studies, will help identify the main factors involved in permissiveness to POMS. It will also be necessary to evaluate the relative weights of, and interactions between, these factors during pathogenesis. This can be achieved through the study of different markers (including oyster genes, viral and bacterial load and genes, *etc.*) identified in real farming conditions with oysters of different ages and genetic backgrounds in different environmental conditions and under different physiological conditions. Such studies are necessary for the implementation of predictive models of the epidemiological risk of POMS.

Theoretical approaches to the study of bivalve infections are still in their infancy (97). Moreover, only a few epidemiological models have been developed for multiple infections, and these models usually oversimplify within-host dynamics (98). Consequently, an additional research objective could be the development of models that synthetically represent POMS under the influence of relevant host, pathogen, and environmental factors. Importantly, these models will help quantify the relative benefits and risks of changes in oyster farming practices (e.g. provision of food resources, age of the oysters at the time of transfer from the bio-secured hatcheries to the natural farming environment).

To conclude, we cannot exclude that other factors could influence the disease. As an example, we can mention field studies indicating that POMS could be influenced by the presence of particles (*e.g.* plankton) carrying OsHV-1 (90, 99–101). Future studies will focus on these factors and their weight in POMS expression, and they can be included in the proposed modeling approaches to get a comprehensive view of POMS and predict as faithfully as possible the epidemiological risk.

## Author Contributions

DD-G, BP, and FP contributed equally to the content of the review and figures. JD, ET, and LD contributed to the content of the review. GM supervised, edited, and added references in the review. All authors contributed to the article and approved the submitted version.

## Funding

The present study was supported by the ANR projects DECIPHER (ANR-14-CE19-0023) and DECICOMP (ANR-19-CE20-0004), and by Ifremer, CNRS, Université de Montpellier, and Université de Perpignan *via* Domitia. This study is set within the framework of the “Laboratoires d’Excellence (LABEX)” TULIP (ANR-10-LABX-41) and CEMEB (ANR-10-LABX-04-01).

## Conflict of Interest

The authors declare that the research was conducted in the absence of any commercial or financial relationships that could be construed as a potential conflict of interest.

## References

[1] FAO. (2015). https://ourworldindata.org/grapher/capture-fisheries-vs-aquaculture-farmed-fish-production.

[2] Ruesink JL Lenihan HS Trimble AC Heiman KW Micheli F Byers JE. Introduction of Non-Native Oysters: Ecosystem Effects and Restoration Implications. Annu Rev Ecology Evol System (2005) 36(1):643–89. 10.1146/annurev.ecolsys.36.102003.152638

[3] FAO. Fishery and aquaculture statistics. Rome: FAO yearbook (2014).

[4] Samain J-F Mc Combie H. Summer mortality of Pacific oyster Crassostrea gigas. MOREST Project (2008).

[5] ECOSCOPA. (2020). https://wwz.ifremer.fr/observatoire_conchylicole/.

[6] Fleury E Barbier P Petton B Normand J Thomas Y Pouvreau S. Latitudinal drivers of oyster mortality: deciphering host, pathogen and environmental risk factors. Sci Rep (2020) 10(1):7264. 10.1038/s41598-020-64086-1 32350335 PMC7190702

[7] Paul-Pont I Dhand NK Whittington RJ. Influence of husbandry practices on OsHV-1 associated mortality of Pacific oysters Crassostrea gigas. Aquaculture (2013) 412:202–14. 10.1016/j.aquaculture.2013.07.038

[8] Barbosa Solomieu V Renault T Travers MA. Mass mortality in bivalves and the intricate case of the Pacific oyster, Crassostrea gigas. J Invertebr Pathol (2015) 131:2–10. 10.1016/j.jip.2015.07.011 26210497

[9] EFSA PoAHW. Oyster mortality. EFSA J (2015) 13(6):4122–n/a. 10.2903/j.efsa.2015.4122

[10] Azéma P Lamy JB Boudry P Renault T Travers MA Dégremont L. Genetic parameters of resistance to Vibrio aestuarianus, and OsHV-1 infections in the Pacific oyster, Crassostrea gigas, at three different life stages. Genet Selection Evol (2017) 49:1–16. 10.1186/s12711-017-0297-2 PMC5311879 28201985

[11] Delisle L Pauletto M Vidal-Dupiol J Petton B Bargelloni L Montagnani C. High temperature induces transcriptomic changes in Crassostrea gigas that hinders progress of Ostreid herpesvirus (OsHV-1) and promotes survival. J Exp Biol (2020) 223(20):1–11. 10.1242/jeb.226233 PMC7578350 32816959

[12] Le Roux F Wegner KM Polz MF. Oysters and Vibrios as a Model for Disease Dynamics in Wild Animals. Trends Microbiol (2016) 24(7):568–80. 10.1016/j.tim.2016.03.006 27038736

[13] Pernet F Barret J Le Gall P Corporeau C Dégremont L Lagarde F. Mass mortalities of Pacific oysters Crassostrea gigas reflect infectious diseases and vary with farming practices in the Mediterranean Thau lagoon, France. Aquaculture Environ Interact (2012) 2(3):215–37. 10.3354/aei00041

[14] Pernet F Tamayo D Fuhrmann M Petton B. Deciphering the effect of food availability, growth and host condition on disease susceptibility in a marine invertebrate. J Exp Biol (2019) 222(Pt 17):1–6. 10.1242/jeb.210534 31439650

[15] Petton B Bruto M James A Labreuche Y Alunno-Bruscia M Le Roux F. Crassostrea gigas mortality in France: the usual suspect, a herpes virus, may not be the killer in this polymicrobial opportunistic disease. Front Microbiol (2015) 6:686. 10.3389/fmicb.2015.00686 26217318 PMC4491618

[16] Petton B Pernet F Robert R Boudry P. Temperature influence on pathogen transmission and subsequent mortalities in juvenile Pacific oysters Crassostrea gigas. Aquaculture Environ Interact (2013) 3(3):257–73. 10.3354/aei00070

[17] Segarra A Pepin JF Arzul I Morga B Faury N Renault T. Detection and description of a particular Ostreid herpesvirus 1 genotype associated with massive mortality outbreaks of Pacific oysters, Crassostrea gigas, in France in 2008. Virus Res (2010) 153(1):92–9. 10.1016/j.virusres.2010.07.011 20638433

[18] Martenot C Oden E Travaille E Malas JP Houssin M. Detection of different variants of Ostreid Herpesvirus 1 in the Pacific oyster, Crassostrea gigas between 2008 and 2010. Virus Res (2011) 160(1-2):25–31. 10.1016/j.virusres.2011.04.012 21600247

[19] Renault T Moreau P Faury N Pepin JF Segarra A Webb S. Analysis of clinical ostreid herpesvirus 1 (Malacoherpesviridae) specimens by sequencing amplified fragments from three virus genome areas. J Virol (2012) 86(10):5942–7. 10.1128/JVI.06534-11 PMC3347280 22419803

[20] Peeler EJ Reese RA Cheslett DL Geoghegan F Power A Thrush MA. Investigation of mortality in Pacific oysters associated with Ostreid herpesvirus-1 muVar in the Republic of Ireland in 2009. Prev Vet Med (2012) 105(1-2):136–43. 10.1016/j.prevetmed.2012.02.001 22398251

[21] Lynch SA Carlsson J Reilly AO Cotter E Culloty SC. A previously undescribed ostreid herpes virus 1 (OsHV-1) genotype detected in the pacific oyster, Crassostrea gigas, in Ireland. Parasitology (2012) 139(12):1526–32. 10.1017/S0031182012000881 23036593

[22] Abbadi M Zamperin G Gastaldelli M Pascoli F Rosani U Milani A. Identification of a newly described OsHV-1 microvar from the North Adriatic Sea (Italy). J Gen Virol (2018) 99(5):693–703. 10.1099/jgv.0.001042 29580370 PMC5994699

[23] Burioli EAV Prearo M Houssin M. Complete genome sequence of Ostreid herpesvirus type 1 microVar isolated during mortality events in the Pacific oyster Crassostrea gigas in France and Ireland. Virology (2017) 509:239–51. 10.1016/j.virol.2017.06.027 28672223

[24] Corbeil S Faury N Segarra A Renault T. Development of an in situ hybridization assay for the detection of ostreid herpesvirus type 1 mRNAs in the Pacific oyster, Crassostrea gigas. J Virol Methods (2015) 211:43–50. 10.1016/j.jviromet.2014.10.007 25455903

[25] Pepin JF Riou A Renault T. Rapid and sensitive detection of ostreid herpesvirus 1 in oyster samples by real-time PCR. J Virol Methods (2008) 149(2):269–76. 10.1016/j.jviromet.2008.01.022 18342377

[26] Renault T Tchaleu G Faury N Moreau P Segarra A Barbosa-Solomieu V. Genotyping of a microsatellite locus to differentiate clinical Ostreid herpesvirus 1 specimens. Vet Res (2015) 45:3. 10.1186/1297-9716-45-3 PMC3897894 24410800

[27] Bruto M James A Petton B Labreuche Y Chenivesse S Alunno-Bruscia M. Vibrio crassostreae, a benign oyster colonizer turned into a pathogen after plasmid acquisition. ISME J (2017) 11(4):1043–52. 10.1038/ismej.2016.162 PMC5364345 27922600

[28] Bruto M Labreuche Y James A Piel D Chenivesse S Petton B. Ancestral gene acquisition as the key to virulence potential in environmental Vibrio populations. ISME J (2018) 12(12):2954–66. 10.1038/s41396-018-0245-3 PMC6246604 30072747

[29] Petton B de Lorgeril J Mitta G Daigle G Pernet F Alunno-Bruscia M. Fine-scale temporal dynamics of herpes virus and vibrios in seawater during a polymicrobial infection in the Pacific oyster Crassostrea gigas. Dis Aquat Organ (2019) 135(2):97–106. 10.3354/dao03384 31342911

[30] de Lorgeril J Lucasson A Petton B Toulza E Montagnani C Clerissi C. Immune-suppression by OsHV-1 viral infection causes fatal bacteraemia in Pacific oysters. Nat Commun (2018) 9(1):4215. 10.1038/s41467-018-06659-3 30310074 PMC6182001

[31] Xiong A Clarke-Katzenberg RH Valenzuela G Izumi KM Millan MT. Epstein-Barr virus latent membrane protein 1 activates nuclear factor-kappa B in human endothelial cells and inhibits apoptosis. Transplantation (2004) 78(1):41–9. 10.1097/01.tp.0000129805.02631.ef 15257037

[32] Miller LK. An exegesis of IAPs: salvation and surprises from BIR motifs. Trends Cell Biol (1999) 9(8):323–8. 10.1016/s0962-8924(99)01609-8 10407412

[33] Martenot C Gervais O Chollet B Houssin M Renault T. Haemocytes collected from experimentally infected Pacific oysters, Crassostrea gigas: Detection of ostreid herpesvirus 1 DNA, RNA, and proteins in relation with inhibition of apoptosis. PLoS One (2017) 12(5):e0177448. 10.1371/journal.pone.0177448 28542284 PMC5436676

[34] Bai CM Morga B Rosani U Shi J Li C Xin LS. and high-throughput sequencing of Ostreid herpesvirus 1 indicate high genetic diversity and complex evolution process. Virology (2019) 526:81–90. 10.1016/j.virol.2018.09.026 30368056

[35] Burioli EAV Varello K Lavazza A Bozzetta E Prearo M Houssin M. A novel divergent group of Ostreid herpesvirus 1 muVar variants associated with a mortality event in Pacific oyster spat in Normandy (France) in 2016. J Fish Dis (2018) 41(11):1759–69. 10.1111/jfd.12883 30151980

[36] Drake JW Holland JJ. Mutation rates among RNA viruses. Proc Natl Acad Sci USA (1999) 96(24):13910–3. 10.1073/pnas.96.24.13910 PMC24164 10570172

[37] Parvez MK Parveen S. Evolution and Emergence of Pathogenic Viruses: Past, Present, and Future. Intervirology (2017) 60(1-2):1–7. 10.1159/000478729 28772262 PMC7179518

[38] Renner DW Szpara ML. Impacts of Genome-Wide Analyses on Our Understanding of Human Herpesvirus Diversity and Evolution. J Virol (2018) 92(1):1–13. 10.1128/JVI.00908-17 PMC5730764 29046445

[39] Renzette N Pokalyuk C Gibson L Bhattacharjee B Schleiss MR Hamprecht K. Limits and patterns of cytomegalovirus genomic diversity in humans. Proc Natl Acad Sci USA (2015) 112(30):E4120–8. 10.1073/pnas.1501880112 PMC4522815 26150505

[40] Perales C Moreno E Domingo E. Clonality and intracellular polyploidy in virus evolution and pathogenesis. Proc Natl Acad Sci USA (2015) 112(29):8887–92. 10.1073/pnas.1501715112 PMC4517279 26195777

[41] Poirier EZ Vignuzzi M. Virus population dynamics during infection. Curr Opin Virol (2017) 23:82–7. 10.1016/j.coviro.2017.03.013 28456056

[42] Brooke CB. Population Diversity and Collective Interactions during Influenza Virus Infection. J Virol (2017) 91(22):e01164-17. 10.1128/JVI.01164-17 PMC5660503 28855247

[43] Mao ZQ He R Sun M Qi Y Huang YJ Ruan Q. The relationship between polymorphisms of HCMV UL144 ORF and clinical manifestations in 73 strains with congenital and/or perinatal HCMV infection. Arch Virol (2007) 152(1):115–24. 10.1007/s00705-006-0826-8 16896551

[44] Pfeiffer JK Kirkegaard K. Increased fidelity reduces poliovirus fitness and virulence under selective pressure in mice. PLoS Pathog (2005) 1(2):e11. 10.1371/journal.ppat.0010011 16220146 PMC1250929

[45] Xiao Y Rouzine IM Bianco S Acevedo A Goldstein EF Farkov M. RNA Recombination Enhances Adaptability and Is Required for Virus Spread and Virulence. Cell Host Microbe (2016) 19(4):493–503. 10.1016/j.chom.2016.03.009 27078068 PMC4840895

[46] Delmotte J Chaparro C Galinier R de Lorgeril J Petton B Stenger PL. Contribution of Viral Genomic Diversity to Oyster Susceptibility in the Pacific Oyster Mortality Syndrome. Front Microbiol (2020) 11:1579. 10.3389/fmicb.2020.01579 32754139 PMC7381293

[47] Saulnier D De Decker S Haffner P Cobret L Robert M. Garcia C. A large-scale epidemiological study to identify bacteria pathogenic to Pacific oyster Crassostrea gigas and correlation between virulence and metalloprotease-like activity. Microb Ecol (2010) 59(4):787–98. 10.1007/s00248-009-9620-y 20012275

[48] Oyanedel D Labreuche Y Bruto M Amraoui H Robino E Haffner P. Vibrio splendidus O-antigen structure: a trade-off between virulence to oysters and resistance to grazers. Environ Microbiol (2020) 22(10):4264–78. 10.1111/1462-2920.14996 32219965

[49] Lemire A Goudenege D Versigny T Petton B Calteau A Labreuche Y. Populations, not clones, are the unit of vibrio pathogenesis in naturally infected oysters. ISME J (2015) 9(7):1523–31. 10.1038/ismej.2014.233 PMC4478691 25489729

[50] Duperthuy M Schmitt P Garzon E Caro A Rosa RD Le Roux F. Use of OmpU porins for attachment and invasion of Crassostrea gigas immune cells by the oyster pathogen Vibrio splendidus. Proc Natl Acad Sci U S A (2011) 108(7):2993–8. 10.1073/pnas.1015326108 PMC3041119 21282662

[51] Rubio T Oyanedel D Labreuche Y Toulza E Luo X Bruto M. Species-specific mechanisms of cytotoxicity toward immune cells determine the successful outcome of Vibrio infections. Proc Natl Acad Sci U S A (2019) 116(28):14238–47. 10.1073/pnas.1905747116 PMC6628822 31221761

[52] Vanhove AS Rubio TP Nguyen AN Lemire A Roche D Nicod J. Copper homeostasis at the host vibrio interface: lessons from intracellular vibrio transcriptomics. Environ Microbiol (2016) 18(3):875–88. 10.1111/1462-2920.13083 26472275

[53] Go J Deutscher AT Spiers ZB Dahle K Kirkland PD Jenkins C. Mass mortalities of unknown aetiology in Pacific oysters Crassostrea gigas in Port Stephens, New South Wales, Australia. Dis Aquat Organ (2017) 125(3):227–42. 10.3354/dao03146 28792421

[54] Lasa A di Cesare A Tassistro G Borello A Gualdi S Furones D. Dynamics of the Pacific oyster pathobiota during mortality episodes in Europe assessed by 16S rRNA gene profiling and a new target enrichment next-generation sequencing strategy. Environ Microbiol (2019) 21(12):4548–62. 10.1111/1462-2920.14750 PMC7379488 31325353

[55] Lucasson A Luo X Mortaza S de lorgeril J toulza E Petton B. A core of functionally complementary bacteria colonizes oysters in Pacific Oyster Mortality Syndrome. bioRxiv (2020). 10.1101/2020.11.16.384644 PMC10155333 37138356

[56] Destoumieux-Garzon D Canesi L Oyanedel D Travers MA Charriere GM Pruzzo C. Vibrio-bivalve interactions in health and disease. Environ Microbiol (2020). 10.1111/1462-2920.15055 32363732

[57] Piel D Bruto M James A Labreuche Y Lambert C Janicot A. Selection of Vibrio crassostreae relies on a plasmid expressing a type 6 secretion system cytotoxic for host immune cells. Environ Microbiol (2020) 22(10):4198–211. 10.1111/1462-2920.14776 31390475

[58] Harvell CD Mitchell CE Ward JR Altizer S Dobson AP Ostfeld RS. Climate warming and disease risks for terrestrial and marine biota. Science (2002) 296(5576):2158–62. 10.1126/science.1063699 12077394

[59] Ben-Haim Y Zicherman-Keren M Rosenberg E. Temperature-regulated bleaching and lysis of the coral Pocillopora damicornis by the novel pathogen Vibrio coralliilyticus. Appl Environ Microbiol (2003) 69(7):4236–42. 10.1128/AEM.69.7.4236-4242.2003 PMC165124 12839805

[60] Ittiprasert W Knight M. Reversing the resistance phenotype of the Biomphalaria glabrata snail host Schistosoma mansoni infection by temperature modulation. PLoS Pathog (2012) 8(4):e1002677. 10.1371/journal.ppat.1002677 22577362 PMC3343117

[61] Kimes NE Grim CJ Johnson WR Hasan NA Tall BD Kothary MH. Temperature regulation of virulence factors in the pathogen Vibrio coralliilyticus. ISME J (2012) 6(4):835–46. 10.1038/ismej.2011.154 PMC3309362 22158392

[62] Vidal-Dupiol J Dheilly NM Rondon R Grunau C Cosseau C Smith KM. Thermal stress triggers broad Pocillopora damicornis transcriptomic remodeling, while Vibrio coralliilyticus infection induces a more targeted immuno-suppression response. PLoS One (2014) 9(9):e107672. 10.1371/journal.pone.0107672 25259845 PMC4178034

[63] Vidal-Dupiol J Ladriere O Destoumieux-Garzon D Sautiere PE Meistertzheim AL Tambutte E. Innate immune responses of a scleractinian coral to vibriosis. J Biol Chem (2011) 286(25):22688–98. 10.1074/jbc.M110.216358 PMC3121412 21536670

[64] Pernet F Lagarde F Jeannee N Daigle G Barret J Le Gall P. Spatial and temporal dynamics of mass mortalities in oysters is influenced by energetic reserves and food quality. PLoS One (2014) 9(2):e88469. 10.1371/journal.pone.0088469 24551106 PMC3925110

[65] Petton B Boudry P Alunno-Bruscia M Pernet F. Factors influencing disease-induced mortality of Pacific oysters Crassostrea gigas. Aquaculture Environ Interact (2015) 6(3):205–22. 10.3354/aei00125

[66] Delisle L Petton B Burguin JF Morga B Corporeau C Pernet F. Temperature modulate disease susceptibility of the Pacific oyster Crassostrea gigas and virulence of the Ostreid herpesvirus type 1. Fish Shellfish Immunol (2018) 80:71–9. 10.1016/j.fsi.2018.05.056 29859311

[67] Dégremont L. Evidence of herpesvirus (OsHV-1) resistance in juvenile Crassostrea gigas selected for high resistance to the summer mortality phenomenon. Aquaculture (2011) 317(1-4):94–8. 10.1016/j.aquaculture.2011.04.029

[68] Segarra A Mauduit F Faury N Trancart S Degremont L Tourbiez D. Dual transcriptomics of virus-host interactions: comparing two Pacific oyster families presenting contrasted susceptibility to ostreid herpesvirus 1. BMC Genomics (2014) 15(580):1–13. 10.1186/1471-2164-15-580 25012085 PMC4111845

[69] Divilov K Schoolfield B Morga B Dégremont L Burge CA Mancilla Cortez D. First evaluation of resistance to both a California OsHV-1 variant and a French OsHV-1 microvariant in Pacific oysters. BMC Genet (2019) 20(1):96. 10.1186/s12863-019-0791-3 31830898 PMC6909534

[70] Camara MD Yen S Kaspar HF Kesarcodi-Watson A King N Jeffs AG. Assessment of heat shock and laboratory virus challenges to selectively breed for ostreid herpesvirus 1 (OsHV-1) resistance in the Pacific oyster, Crassostrea gigas. Aquaculture (2017) 469:50–8. 10.1016/j.aquaculture.2016.11.031

[71] Dégremont L Nourry M Maurouard E. Mass selection for survival and resistance to OsHV-1 infection in Crassostrea gigas spat in field conditions: response to selection after four generations. Aquaculture (2015) 446:111–21. 10.1016/j.aquaculture.2015.04.029

[72] Divilov K Schoolfield B Morga B Degremont L Burge CA Mancilla Cortez D. First evaluation of resistance to both a California OsHV-1 variant and a French OsHV-1 microvariant in Pacific oysters. BMC Genet (2019) 20(1):96. 10.1186/s12863-019-0791-3 31830898 PMC6909534

[73] Zhang G Fang X Guo X Li L Luo R Xu F. The oyster genome reveals stress adaptation and complexity of shell formation. Nature (2012) 490(7418):49–54. 10.1038/nature11413 22992520

[74] Gutierrez AP Turner F Gharbi K Talbot R Lowe NR Penaloza C. Development of a Medium Density Combined-Species SNP Array for Pacific and European Oysters (Crassostrea gigas and Ostrea edulis). G3 (Bethesda) (2017) 7(7):2209–18. 10.1534/g3.117.041780 PMC5499128 28533337

[75] Gutierrez AP Bean TP Hooper C Stenton CA Sanders MB Paley RK. A Genome-Wide Association Study for Host Resistance to Ostreid Herpesvirus in Pacific Oysters (Crassostrea gigas). G3 (Bethesda) (2018) 8(4):1273–80. 10.1534/g3.118.200113 PMC5873916 29472307

[76] de Lorgeril J Petton B Lucasson A Perez V Stenger PL Degremont L. Differential basal expression of immune genes confers Crassostrea gigas resistance to Pacific oyster mortality syndrome. BMC Genomics (2020) 21(1):63. 10.1186/s12864-020-6471-x 31959106 PMC6971885

[77] Oldenburg M Kruger A Ferstl R Kaufmann A Nees G Sigmund A. TLR13 recognizes bacterial 23S rRNA devoid of erythromycin resistance-forming modification. Science (2012) 337(6098):1111–5. 10.1126/science.1220363 22821982

[78] Shi Z Cai Z Sanchez A Zhang T Wen S Wang J. A novel Toll-like receptor that recognizes vesicular stomatitis virus. J Biol Chem (2011) 286(6):4517–24. 10.1074/jbc.M110.159590 PMC3039399 21131352

[79] Oden E Martenot C Berthaux M Travaillé E Malas JP Houssin M. Quantification of ostreid herpesvirus 1 (OsHV-1) in Crassostrea gigas by real-time PCR: Determination of a viral load threshold to prevent summer mortalities. Aquaculture (2011) 317(1-4):27–31. 10.1016/j.aquaculture.2011.04.001

[80] Dégremont L. Size and genotype affect resistance to mortality caused by OsHV-1 in Crassostrea gigas. Aquaculture (2013) 416-417:129–34. 10.1016/j.aquaculture.2013.09.011

[81] Lochmiller RL Deerenberg C. Trade-offs in evolutionary immunology: just what is the cost of immunity? Oikos (2000) 88(1):87–98. 10.1034/j.1600-0706.2000.880110.x

[82] Sheldon BC Verhulst S. Ecological immunology: costly parasite defences and trade-offs in evolutionary ecology. Trends Ecol Evol (1996) 11(8):317–21. 10.1016/0169-5347(96)10039-2 21237861

[83] Smith VH Jones TP Smith MS. Host nutrition and infectious disease: an ecological view. Front Ecol Environ (2005) 3(5):268–74. 10.1890/1540-9295(2005)003[0268:HNAIDA]2.0.CO;2

[84] Civitello DJ Fatima H Johnson LR Nisbet RM Rohr JR. Bioenergetic theory predicts infection dynamics of human schistosomes in intermediate host snails across ecological gradients. Ecol Lett (2018) 21(5):692–701. 10.1111/ele.12937 29527787

[85] Hall SR Simonis JL Nisbet RM Tessier AJ Cáceres CE. Resource Ecology of Virulence in a Planktonic Host-Parasite System: An Explanation Using Dynamic Energy Budgets. Am Nat (2009) 174(2):149–62. 10.1086/600086 19527119

[86] Ayres JS Schneider DS. The role of anorexia in resistance and tolerance to infections in Drosophila. PLoS Biol (2009) 7(7):e1000150. 10.1371/journal.pbio.1000150 19597539 PMC2701602

[87] Murray MJ Murray AB. Anorexia of infection as a mechanism of host defense. Am J Clin Nutr (1979) 32(3):593–6. 10.1093/ajcn/32.3.593 283688

[88] Evans O Hick P Dhand N Whittington RJ. Transmission of Ostreid herpesvirus-1 in Crassostrea gigas by cohabitation: effects of food and number of infected donor oysters. Aquaculture Env Interact (2015) 7(3):281–95. 10.3354/aei00160

[89] Moreau P Moreau K Segarra A Tourbiez D Travers MA Rubinsztein DC. Autophagy plays an important role in protecting Pacific oysters from OsHV-1 and Vibrio aestuarianus infections. Autophagy (2015) 11(3):516–26. 10.1080/15548627.2015.1017188 PMC4502751 25714877

[90] Pernet F Fuhrmann M Petton B Mazurie J Bouget JF Fleury E. Determination of risk factors for herpesvirus outbreak in oysters using a broad-scale spatial epidemiology framework. Sci Rep (2018) 8(1):10869. 10.1038/s41598-018-29238-4 30022088 PMC6052024

[91] Jouaux A Lafont M Blin J-L Houssin M Mathieu M Lelong C. Physiological change under OsHV-1 contamination in Pacific oyster *Crassostrea gigas* through massive mortality events on fields. BMC Genomics (2013) 14(590):1–14. 10.1186/1471-2164-14-590 23987141 PMC3766697

[92] Segarra A Mauduit F Faury N Trancart S Dégremont L Tourbiez D. Dual transcriptomics of virus-host interactions: Comparing two Pacific oyster families presenting contrasted susceptibility to ostreid herpesvirus 1. BMC Genomics (2014) 15(1):1–13. 10.1186/1471-2164-15-580 25012085 PMC4111845

[93] Desai M Fang R Sun J. The role of autophagy in microbial infection and immunity. ImmunoTargets Ther (2015) 4:13–26. 10.2147/ITT.S76720 27471708 PMC4918246

[94] Wegner KM Volkenborn N Peter H Eiler A. Disturbance induced decoupling between host genetics and composition of the associated microbiome. BMC Microbiol (2013) 13:252. 10.1186/1471-2180-13-252 24206899 PMC3840651

[95] King WL Siboni N Williams NLR Kahlke T Nguyen KV Jenkins C. Variability in the Composition of Pacific Oyster Microbiomes Across Oyster Families Exhibiting Different Levels of Susceptibility to OsHV-1 muvar Disease. Front Microbiol (2019) 10:473. 10.3389/fmicb.2019.00473 30915058 PMC6421512

[96] Clerissi C de Lorgeril J Petton B Lucasson A Escoubas JM Gueguen Y. Microbiota Composition and Evenness Predict Survival Rate of Oysters Confronted to Pacific Oyster Mortality Syndrome. Front Microbiol (2020) 11:311. 10.3389/fmicb.2020.00311 32174904 PMC7056673

[97] Pernet F Lupo C Bacher C Whittington RJ. Infectious diseases in oyster aquaculture require a new integrated approach. Philos Trans R Soc Lond B Biol Sci (2016) 371(1689):1–9. 10.1098/rstb.2015.0213 PMC4760143 26880845

[98] Sofonea MT Alizon S Michalakis Y. Exposing the diversity of multiple infection patterns. J Theor Biol (2017) 419:278–89. 10.1016/j.jtbi.2017.02.011 28193485

[99] Garcia C Thebault A Degremont L Arzul I Miossec L Robert M. Ostreid herpesvirus 1 detection and relationship with Crassostrea gigas spat mortality in France between 1998 and 2006. Vet Res (2011) 42:73. 10.1186/1297-9716-42-73 21635731 PMC3129302

[100] Paul-Pont I Dhand NK Whittington RJ. Spatial distribution of mortality in Pacific oysters Crassostrea gigas: reflection on mechanisms of OsHV-1 transmission. Dis Aquat Organ (2013) 105(2):127–38. 10.3354/dao02615 23872856

[101] Whittington RJ Liu O Hick PM Dhand N Rubio A. Long-term temporal and spatial patterns of Ostreid herpesvirus 1 (OsHV-1) infection and mortality in sentinel Pacific oyster spat (Crassostrea gigas) inform farm management. Aquaculture (2019) 513:1–15. 10.1016/j.aquaculture.2019.734395

